# Investigation of the Impact of School Climate and Teachers’ Self-Efficacy on Job Satisfaction: A Cross-Cultural Approach

**DOI:** 10.3390/ejihpe10010011

**Published:** 2019-10-14

**Authors:** Ioannis G. Katsantonis

**Affiliations:** Department of Primary Education, University of Patras, 265 04 Patras, Greece; ioanniskatsantonis@upnet.gr

**Keywords:** teachers’ self-efficacy, job satisfaction, school climate, primary schools, TALIS 2018, OECD, structural equation modeling, general linear model, mediation analysis, cross-cultural research

## Abstract

The purpose of this study is twofold: (a) To confirm the mediating role of teachers’ self-efficacy between the relation of school climate and teachers’ job satisfaction and (b) to tease apart any cross-cultural effects of the association of self-efficacy and job satisfaction by comparing teachers’ responses. Drawing upon the publicly available TALIS 2018 (June 2019) database, a representative sample of 51,782 primary school teachers from 15 countries was used for the analyses. Structural equation modeling was implemented to test for mediation effects of teachers’ self-efficacy at the individuals’ level and a general linear model (GLM) MANOVA was applied to compare the participants’ scores in self-efficacy and job satisfaction across cultures. Results indicate, in accordance with previous research, that self-efficacy is a mediating variable of the relation between school climate and job satisfaction at the individuals’ level across cultures. Moreover, the GLM revealed statistically significant cross-cultural differences among teachers’ responses in job satisfaction and self-efficacy. These findings have implications for teachers’ wellbeing and resilience.

## 1. Introduction

Teachers’ self-efficacy and job satisfaction are two emerging fields of interest to applied researchers [1]. The construct of teachers’ self-efficacy is closely connected with school climate variables such as teachers’ perception of students’ behavior. These school context factors are described, in the international literature, as variables that influence teachers’ self-efficacy (cf., [1,2]). Further, teachers’ self-efficacy beliefs are also postulated as mediators between school climate and job satisfaction dimensions [1,3]. Large scale literature reviews have suggested that school climate contributes significantly, among other factors, to students’ achievement, teachers’ intention to remain professionally active, and successful school environment [4]. Thus, school climate has been recognized by teachers as an essential part of the schools [5] and by researchers as a central point of applied research interest [6].

Another variable of interest is teachers’ job satisfaction. Teachers’ job satisfaction has proven to be very interesting due to the fact that it is associated indirectly with students’ academic achievement and directly with teachers’ wellbeing in the form of psychological resilience and prevention of burnout, etc. [7]. Lastly, teachers’ self-efficacy and teachers’ job satisfaction have been compared in cross-cultural settings with significant differences found among culturally diverse samples (cf., [8,9,10]).

As can be seen from a first glimpse of the wider field, the three variables of interest are interconnected. Despite the above, few studies have extensively investigated the cross-cultural effects of these constructs and their relations. The literature that concerns the mediating role of teachers’ self-efficacy between the relationship of school climate and job satisfaction at the individual level is also, to our knowledge, quite limited. Furthermore, few studies have jointly compared and contrasted teachers’ self-efficacy and job satisfaction scores among culturally diverse samples (e.g., [8,9,10]). It is, thereby, sought to jointly address these shortcomings at the ISCED level 1 (i.e., primary school).

### 1.1. Operational Definitions

School climate is a challenging construct to define. Sometimes it is interchangeably referred to as school culture, though, nowadays, school climate is an umbrella term that subsumes school culture (cf., [3,6]). School climate is a multidimensional concept. It is widely accepted that school climate, as a term, includes the following: (a) Schools’ infrastructure (e.g., equipment, etc.), (b) the characteristics of the persons inside schools (i.e., teachers’ and students’ background), (c) the relations among the individuals (e.g., among staff and/or students), (d) the school culture (e.g., attitude, group goals, etc.) [6]. Another study [4] proposes to define school climate as experiences at the individual level (e.g., students’ or staff’s experiences) of the school life, which include values, unwritten social rules (i.e., the acceptable and the non-acceptable), personal purposes, and inter-individual social relations (e.g., the type of teacher-students’ relationship and how the school members feel about each other), etc. Further, school climate is also conceptually linked with human development in a safe school environment (e.g., limited aggression in schools as a result of students’ good behavior), productive learning episodes (e.g., enhancing students’ ability to learn and teachers’ capacity to instruct), and contribution to foster society’s democratic principles. 

On the other hand, teachers’ self-efficacy is also a multidimensional construct. The key literature review by Tschannen-Moran, Woolfolk Hoy, and Hoy [11] conceptualizes teachers’ self-efficacy as teachers’ individual awareness of their effectiveness; that is, the degree of their capacity to motivate students to higher levels of performance. Further, it is noted that teachers’ self-efficacy is a product of Bandura’s socio-cognitive theory and also describes teachers’ efforts to achieve their goals and enhance their personal resilience [12]. As OECD [7] suggests, teachers’ self-efficacy is connected with many teacher-related outcomes. For instance, high self-efficacy scores may help teachers’ retention decisions [7,12], may have a preventive role against burnout syndrome, and overall, high self-efficacy is a factor that facilitates teachers’ wellbeing and resilience.

Teachers’ job satisfaction is a construct of interest in organizational psychology. It refers to teachers’ personal viewpoints and attitudes toward their working environment and their profession in general and is critical for teachers’ psychological wellbeing (cf., [7,13]). An additional theoretical view of job satisfaction is traced to the much-cited article of Locke [14]. He describes job satisfaction as an affectional situation that is created from individuals’ own critical performance in the job or the employees’ promotion of the job’s values (p. 316). Job satisfaction is considered a crucial factor in teacher retention [15]. 

### 1.2. School Climate, Teachers’ Self-Efficacy, and Job Satisfaction

Collie, Shapka, and Perry [1] have implemented a structural equation modeling (SEM) approach with a sample of 664 (secondary and elementary) teachers from Canada. Their model identified school climate variables to significantly impact teachers’ job satisfaction. Further, the structural model distinguished statistically significant indirect effects, i.e., teachers’ self-efficacy acted as a mediator between school climate and job satisfaction. Another similar research is that of Aldridge and Fraser [3], who also conducted SEM analysis with 781 high school teachers. Their findings indicate that school climate variables were directly affecting job satisfaction and teachers’ self-efficacy was established as a mediating variable. Moreover, it should be noted that other research findings [16] have studied teachers’ self-efficacy and collective efficacy in conjunction with job satisfaction and school climate variables (e.g., the perceived behavior of colleagues, principals, and students; teachers-parents’ collaboration, etc.) but have not tested for any specific indirect effects between school climate variables and job satisfaction. A more recent study is that of Malinen and Savolainen [17]. They used a sample of 642 Finnish secondary school teachers to assess the direct effect of school climate on self-efficacy and then to test for a possible mediating effect of self-efficacy between school climate and job satisfaction and between school climate and burnout. Their results showed both a positive direct effect of school climate on self-efficacy (β = 0.26) and an indirect effect of school climate through self-efficacy on job satisfaction (β = 0.08). Although the study of Taylor and Tashakkori [18] is older than the aforementioned, it has established with a sample of 9987 teachers that teachers’ sense of self-efficacy has an impact on job satisfaction and that school climate strongly influences job satisfaction and moderately impacts self-efficacy. It is therefore underlined that teachers’ self-efficacy could either possibly directly affect job satisfaction or act as a mediating variable between school climate and job satisfaction.

### 1.3. Cross-Cultural Comparisons of Self-Efficacy and Job Satisfaction

A similar study to the proposed one, which is presented in this article, is that of Klassen, Usher, and Bong [8]. These researchers tested for direct effects of two types of teachers’ self-efficacy (i.e., personal and collective), of collectivism (i.e., the focus on the in-group goals and the determination of social behavior based on the group’s needs) and job stress on job satisfaction. What is, though, of utmost importance for the present study is that those authors used teachers’ self-efficacy, collectivism, job stress, and job satisfaction as dependent variables and tested for significant differences (utilizing MANOVA procedures) among their teacher participants (both primary and secondary) from Canada, Korea, and the U.S.A. Their results indicated statistically significant differences, though low effect size *η^2^_partial_* = 0.27, among the culturally diverse samples. More specifically, their results showed that Korean teachers scored lower than all the other groups in all variables; Canadian and U.S.A. teachers had similar ratings. Further, the study by Zieger, Sims, and Jerrim [19] has utilized the TALIS 2013 data to compare UK lower secondary school teachers to the other countries’ teachers. As their analyses indicated, Australia, Israel, New Zealand, Romania, Sweden, U.S.A., Poland, Russia, Brazil, Flanders (Belgium), Italy, Norway, Croatia, and France had displayed higher scores in teachers’ job satisfaction than UK teachers. Lastly, teachers from Slovakia, Latvia, and the Czech Republic had scored about the same as UK teachers.

On the other hand, findings of mean differences across cultures for teachers’ self-efficacy were found by Vieluf, Kunter, and van de Vijver [9], who utilized the previous TALIS 2008 database. Their results indicated that teachers’ self-efficacy was a lot higher for Northern European countries (i.e., Iceland and Denmark), as well as for Anglophone countries (i.e., Ireland and Australia), and in Austria. The Mediterranean and the South American countries (i.e., Spain, Portugal, Brazil, Mexico, and Turkey, with the exception of Italy) displayed low mean self-efficacy scores. The lowest, though, scores were observed from teachers of the Republic of Korea. Additionally, low scores were found in Eastern European countries (i.e., Estonia, Hungary, the Slovak Republic, Poland, Lithuania, Slovenia, and Bulgaria). Another relevant study is that of Klassen et al. [10]. These researchers also focused solely on teachers’ self-efficacy. Their cross-cultural study consisted of 1212 teachers (primary and secondary schools) from five countries, i.e., Canada, Korea, U.S.A., Singapore, and Cyprus. They found (using ANOVAs) that participants from Korea and Singapore had lower self-efficacy scores than the other participants. Concluding, it is essential to emphasize that not all of those studies have jointly considered job satisfaction and teachers’ self-efficacy for cross-cultural comparisons, and neither have they focused solely on the primary school level (ISCED 1).

At this point, it is underscored that in the present study the term “cross-cultural” is not used arbitrarily, but is based on the relevant literature regarding TALIS. First and foremost, OECD [20] in their TALIS 2018 technical report mention that the TALIS project constructs instruments to compare education-related contexts cross-culturally (see pp. 38, 54). Moreover, Vieluf, Kunter, and van de Vijver [9] have found that teachers’ self-efficacy, as measured by OECD’s TALIS program, is connected with culture-specific variables; a finding which is indicative of cross-cultural differentiation. Additionally, the contemporary study by Sun and Xia [21], also carried out with TALIS 2013 data, repeatedly (see pp. 4–5) argues that the job satisfaction and self-efficacy scales could be utilized for cross-cultural comparisons and correlational analyses with other variables. 

### 1.4. The Present Study

**Figure 1:**
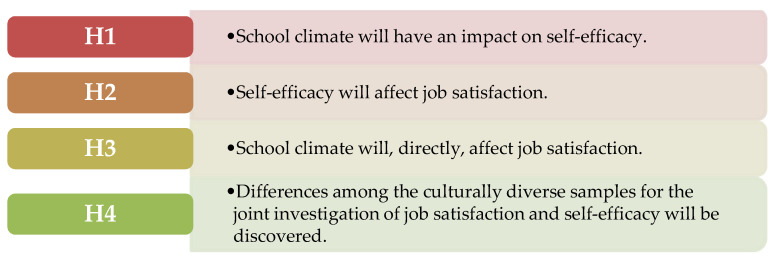
Research hypotheses.

The findings of previous research (e.g., [3,17]) have shown that teachers’ self-efficacy is a mediator between the association of school climate variables and teachers’ job satisfaction. This study is, to our knowledge, the first one to attempt to confirm this claim across culturally diverse settings with an emphasis on ISCED level 1. Prior research has focused exclusively on secondary school teachers (e.g., [3,17]), however, this study focuses solely on ISCED level 1 (primary school teachers). Moreover, studies that have compared primary school teachers’ job satisfaction across cultures are quite limited (e.g., [8,10]; the same could be said about teachers’ self-efficacy (e.g., [8,9]. The majority of those studies have also not taken into consideration the association of job satisfaction and self-efficacy, which could be rectified by jointly using those variables as dependent variables. Subsequently, the following research questions (RQ) and hypotheses (Figure 1) were formulated.

- RQ1: Is it confirmed that self-efficacy mediates the relationship between school climate and teachers’ job satisfaction at the individual level of analysis?
- RQ2: Do primary school teachers differ across cultures regarding their sense of self-efficacy and job satisfaction? If so, how do the cultures differ?

## 2. Method

### 2.1. Data Collection and Participants

The dataset used in this study is a part of the Teaching and Learning International Survey (TALIS 2018) by OECD. Overall, data based on the TALIS 2018 large scale survey project were collected across 48 economies/ countries with two-stage stratified, probabilistic random sampling procedures to ensure the representativeness of the sample [20]. The data collection design, as described by OECD [20], was cross-sectional. The following analyses and the subsequent results are based on the open-access databases that are published by OECD and deposited at https://www.oecd.org/education/talis/talis-2018-data.htm. It should be noted that the TALIS 2018 survey was conducted in late 2017/early 2018 and the initial datasets were published in June 2019. The current study utilized only the ATGINTT3 set, which contains the responses of N = 51,782 primary school teachers (ISCED 1) from 15 countries. The complete sample is divided between 40,965 (79.1%) female and 10,817 (20.9%) male school teachers. All participants’ ages were recorded in age groups as follows: Under 25 years N = 1331 (2.6%); 25 to 29 years N = 6024 (11.6%); 30 to 39 years N = 16,231 (31.3%); 40 to 49 years N = 16,393 (31.7%); 50 to 59 years N = 9843 (19%) and from 60 N = 1928 (3.7%); N = 32 (0.1%) responses were missing. In Table 1 the distribution of the participants across countries is presented.

### 2.2. Measures

All measures, used in the present research, were thoroughly assessed for construct validity and measurement invariance across the culturally diverse samples (for instrument validation see OECD [20]). The interested readership is, also, referred to the TALIS 2018 technical report for all scales’ reliability coefficients across countries regarding the ISCED level 1(see [20]).

#### 2.2.1. Teacher Self-Efficacy 

The three subscales from the widely used teachers’ self-efficacy (TSES) scale [22] were administered. TSES consists of the four-item self-efficacy in instruction subscale (INSTR), the four-item self-efficacy in classroom management subscale (CLMAN), and the four-item self-efficacy in student engagement subscale (STUDENG). The items for each scale are presented in Table 2. All items were anchored with a four point Likert-type scale (“Not at all” (1), “To some extent” (2), “Quite a bit” (3), “A lot” (4)). 

#### 2.2.2. School Climate 

School climate (CLIM) consists of the two available scales, each with four-items length, constructed by the TALIS team. The first subscale represents teachers’ perceived disciplinary climate (DISCP). Another school climate subscale is teacher-student relations (REL). All items were anchored with a four point Likert-type scale (“Strongly disagree” (1), “Disagree” (2), “Agree” (3), “Strongly agree” (4)). The items for each subscale are presented in Table 3.

#### 2.2.3. Job Satisfaction

The two available subscales, each with four-items length, form the overall job satisfaction scale (SATIS). Both instruments were developed by the TALIS team. The subscales are job satisfaction with the work environment and job satisfaction with profession. All items were anchored with a four-point Likert-type scale (“Strongly disagree” (1), “Disagree” (2), “Agree” (3), “Strongly agree” (4)). The items are presented in Table 4.

### 2.3. Missing Data and Data Cleaning

Missing data analysis revealed 6.59% of missing values for the composite (summated) variables. Little’s MCAR test *x*^2^ = 740.67, *df* = 353, *p* < 0.001 (seed 123456) showed that these responses were not missing completely at random. Thus, the expectation-maximization algorithm was applied to impute the missing values. Descriptive statistics for all the composite variables, that were used, are presented in Table A1 in Appendix A. Although multivariate outliers (*N* = 657) were diagnosed utilizing Mahalanobis’ Distance squared, 24.326 < *D*^2^ < 64.263, *p* < 0.001, those values were not excised from the sample as they were thought of as valid response patterns. All statistical procedures presented in this paper were conducted either with IBM SPSS 23.0 [23] or with the Analysis of Moment Structures (AMOS) 23.0 [24] depending on the research objective. For all the analyses an alpha level at 0.01 is assumed.

## 3. Results

### 3.1. Teachers’ Self-Efficacy as a Mediator between School Climate and Job Satisfaction

**Figure 2:**
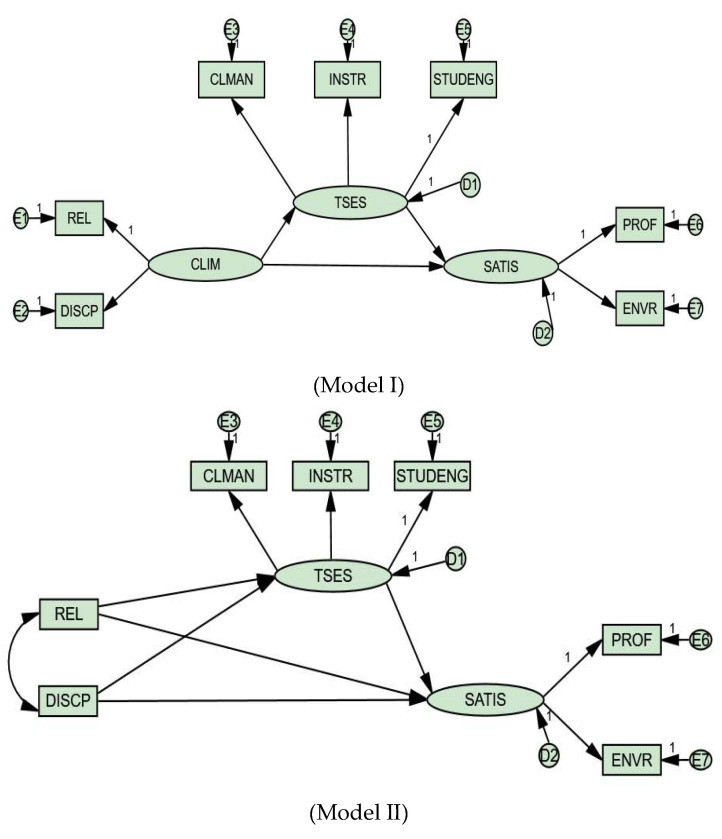
Structural Models: Mediation between school climate and job satisfaction. Note: Please refer to the Measures section for the description of the abbreviations.

Based on the formulated hypotheses H1 to H3, two conceptually similar, but configured differently (Figure 2), structural equation models were developed and tested. It has become apparent (see Measures section) that the school climate scale that pertains to teachers’ perceived disciplinary climate (i.e., DISCP) has a negative intrinsic meaning, and may have a negative effect on teachers’ self-efficacy (i.e., TSES) and job satisfaction (i.e., SATIS). Thus, a second model that includes the separated school climate variables (i.e., REL; DISCP) is also tested to independently assess the effects of those two variables. The pooled sample (*N* = 51,782) across all cultures was utilized in the process, because the level of the analysis is the individual and as has been pointed elsewhere the use of the full sample for individual-level analyses is applicable (see [9]). A two-step SEM approach [25] was implemented. Specifically, first the measurement models (see Table A2 in Appendix A) were specified and then the structural models were fitted to the data covariance matrix. The maximum likelihood (ML) estimator (available in AMOS) will produce biased estimates if the item responses are not at least on a five-point scale (see the landmark study by [26]; see also [27]). The TALIS items are given on a four-point Likert-type scale and thus only composite variables (e.g., DISCP) were used to avoid biased parameter recovery. Further, it is noted that parceling is applied because it produces more normal, continuous indicators that abide by the normal theory ML estimator (cf., [28,29,30]). Lastly, as the present research objective is not measurement validation, the formation of composites from unidimensional scales may be appropriate [28]. Various goodness-of-fit indices were used to assess the models. Based on Hu and Bentler [31] CFI, TLI, AGFI, and GFI values above 0.95 are indicators of an excellent fit. SRMR values lower than 0.05 are also implying excellent model-data fit. RMSEA values between zero and 0.05 are excellent, between 0.05 and 0.08 adequate and above 0.08 mediocre. 

Following the pooled testing of the measurement models, the path models were specified as shown in Figure 2. The structural equation Model I displayed acceptable fit to the sample covariance matrix with the following fit indices’ values: *x^2^(df = 10, N = 51,782)* = 2461.475, *p < 0.001*, *x^2^/df* = 246.147, GFI = 0.98, AGFI = 0.95, TLI = 0.95, CFI = 0.97, RMSEA = 0.07; 90% CI [0.073; 0.077], SRMR = 0.03. The Information Criteria showed the following values: AIC = 3253.10, BCC = 3253.10 and BIC = 3403.63. 

Mardia’s [32] multivariate normality test = 76.19, *p <* 0.001 indicated statistically significant deviation from the multivariate normal distribution; thus, a maximum likelihood non-parametric bootstrap with 1000 draws was applied (see Byrne [33]). Path coefficients, standard errors, and the bootstrapping results are presented in Table 5.

The indirect effect of *school climate* on *job satisfaction* is 0.563 × (−0.282) = −0.158, *p <* 0.001; 90% CI [−0.181; −0.140] and the direct effect of *school climate* on *job satisfaction* is 0.92. Model II, though, displayed better fit than Model I. The goodness-of-fit indices and the Information Criteria were as follows: *x^2^(df* = 10, *N* = 51,782) = 2461.475, *p* < 0.001, *x^2^/df* = 246.147, GFI = 0.98, AGFI = 0.96, TLI = 0.95, CFI = 0.98, RMSEA = 0.06; 90% CI [0.067; 0.071], SRMR = 0.02, AIC = 2497.47, BCC = 2497.48, and BIC = 2656.86. Path coefficients, standard errors, and the bootstrapping results for Model II are presented in Table 6.

The indirect effect of *teachers’ perceived disciplinary climate* on *job satisfaction* is −0.02, *p <* 0.001; 90% CI [−0.25; −0.20]. The indirect effect of *teacher-student relations* on *job satisfaction* is 0.02, *p <* 0.001; 90% CI [0.020; 0.024]. These findings are discussed more in detail in the Discussion section. 

### 3.2. Cross-Cultural Comparisons of Teachers’ Self-Efficacy and Job Satisfaction

As a foreword for the present section, it is noted that the analytical approach of latent mean differences (LMD) is a more elegant method to test for between-group differences in psychological research, but is dependent on a few statistical assumptions (cf., [27,33,34,35]. Measurement invariance and specifically scalar invariance (i.e., intercepts constrained to equal) is a prerequisite (cf., [27,33,34,35]. In the OECD TALIS 2018 technical report [20] configural (i.e., equal factor structure) and metric invariance (i.e., equal loadings) have been achieved, but scalar invariance was not established (see pp. 276 for self-efficacy scales; 294 for job satisfaction scales) and an LMD approach is not applicable. Thus, the less restrictive MANOVA procedure is applied, as was done in previous cross-cultural research (e.g., [8,10]. 

For RQ2 the general linear model was utilized to conduct a one-way MANOVA. The analysis was conducted using the aggregated teachers’ self-efficacy subscales and the aggregated job satisfaction subscales as dependent variables. As independent variable teachers’ classification based on geographical location was utilized (see Table 1). Teachers’ self-efficacy and job satisfaction were introduced to the model due to empirical and theoretical reasons, as it is appropriate (cf., [36, 37, 38]. In this study, they are correlated variables (*r =* 0.213, *p <* 0.001) and previous research have studied them jointly (e.g., [8]). 

Mardia’s multivariate normality test [32] for the composite variables (see descriptive statistics in Appendix A, Table A1) indicated a statistically significant departure from multivariate normal distribution (8.56, *p* < 0.001). However, the MANOVA procedure is quite robust to non-normality (e.g., [36]). The results of the omnibus multivariate tests provide significant statistical evidence that the fifteen groups’ mean centroids differ, Wilk’s *Λ* = 0.66, *F (28, 51,782) =* 837.83, *p <* 0.001; *η^2^_Mult._* = 0.33 (for the calculation of eta multivariate see [38]). Similarly, statistically significant results at *p <* 0.001 are supported by the other multivariate test criteria (Table A3 in Appendix A). Both of the univariate tests were, also, highly significant with job satisfaction *F(14, 51,782) =* 378.57, *p <* 0.001, partial *η^2^* = 0.09; and teachers’ self-efficacy *F(14, 51,782) =* 1312.37, *p <* 0.001, partial *η^2^* = 0.26. 

**Figure 3:**
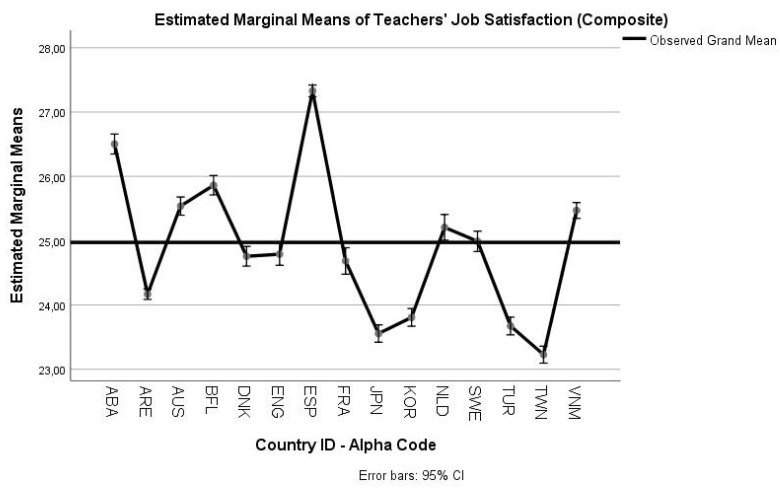
Profile plot for teachers’ job satisfaction for subsamples. Note: ABA1: Chile; ARE1: United Arab Emirates; AUS1: Australia; BFL1: Flemish Community (Belgium); DNK1: Denmark; ENG1: UK; ESP1: Spain; FRA1: France; JPN1: Japan; KOR1: Korea; NLD1: Netherlands; SWE1: Sweden; TUR1: Turkey; TWN1: Chinese Taipei; VNM1: Viet Nam.

**Figure 4:**
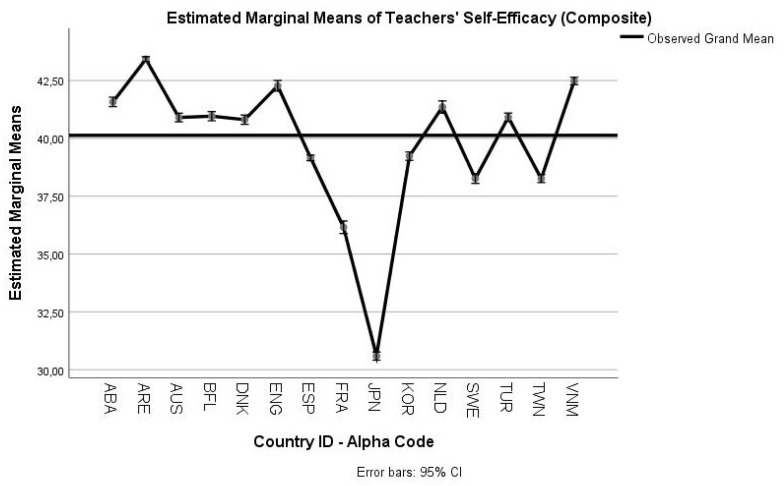
Profile plot for teachers’ self-efficacy for subsamples.

In Figure 3 the profile plot for the job satisfaction variable is presented. Figure 4 depicts the profile plot for the teachers’ self-efficacy variable (see descriptive statistics for each dependent variable in Table A4, Appendix A).

## 4. Discussion

### 4.1. The Contribution of the Study

The present approach sought to explore two research questions, i.e., whether teachers’ self-efficacy could be supported as a mediating variable between school climate and job satisfaction at the individual level of analysis, and whether there are significant cross-cultural differences among the participants in their self-efficacy and job satisfaction scores. We were able to identify only one study (i.e., [3] to have completely focused on the mediating role of self-efficacy between the relation of school climate and job satisfaction and one other study (i.e., [17]) which has examined that mediating effect in conjunction, though, with burnout. Another study has contemplated that association within a broader model of school climate and social-emotional learning [1] and one other has used simple regressions without testing for mediation effects (i.e., [18]). However, neither study has had a cross-cultural approach with a broad range of cultural diversity as the present one. Additionally, a cross-cultural comparison of teachers’ self-efficacy and job satisfaction has been conducted either with only one variable (i.e., self-efficacy) (e.g., [9,10,19]) or with multiple variables (e.g., [8] that may confound the joint association between teachers’ self-efficacy and job satisfaction. 

### 4.2. Interpretation of the Results

As it is the current trend to test for multiple models [25], two structural equation models were tested. The first model (Model I) compared to the second (Model II) was less well-fitting to the data covariance matrix. Thus, Model II is thought to reflect better the inter-relationships among the variables. As shown in Model II (see Table 6) the school climate variables (i.e., teachers’ perceived disciplinary climate and teacher-student relationship) influence teachers’ self-efficacy. Thus, Hypothesis 1 (H1) is confirmed. The first school climate variable (i.e., teachers’ perceived disciplinary climate) has a negative meaning and therefore a negative impact on teachers’ self-efficacy. The second variable (i.e., teacher-student relationship) has a positive intrinsic meaning and thus a positive influence on teachers’ self-efficacy. These results are in accordance with previous studies (e.g., [1,3,17]), which have indicated that school climate variables affect teachers’ self-efficacy. Beyond the matter of the influence of school climate on self-efficacy, it should also be mentioned that teachers’ self-efficacy beliefs were found to mediate the association between school climate and job satisfaction. Thus, Hypotheses 2 and 3 (H2–3) are confirmed. For Modell II the indirect effects were rather weak (β = 0.02; β = −0.02, *p < 0.001)*, but the indirect effect for Model I was stronger (β = −0.15, *p < 0.001*). We hypothesize that the discrepancy between the magnitude of the indirect effects of the two models is because in Model I the measurement errors of the school climate variables are taken directly into consideration (i.e., the model is fully latent). These results comply with the studies conducted by Aldridge and Fraser [3], Collie, Shapka, and Perry [1], and Malinen and Savolainen [17]. It is underlined that it was expected for this mediation, though partial in nature, to occur as both job satisfaction and teachers’ self-efficacy were predicted by school climate, and job satisfaction was also predicted by self-efficacy in a previous study [18].

Regarding the RQ2, the findings of the analyses also complied with prior empirical studies. The general linear model indicated significant mean differences in teachers’ self-efficacy and job satisfaction across cultures. Thus, Hypothesis 4 (H4) is confirmed. This result is in accordance with the findings of Klassen, Usher, and Bong [8], who argued for statistically significant differences among their Canadian, Korean, and U.S.A. participants. The cross-cultural differentiation of job satisfaction is also in line with the findings of Zieger, Sims, and Jerrim [19]. Although the study by Vieluf, Kunter, and van de Vijver [9] did not examine job satisfaction at all, they found significant mean differences across-cultures for teachers’ self-efficacy. Further, the study by Klassen et al. [10] also focused solely on teachers’ self-efficacy with similar results with the aforementioned differences. A brief study of the profile plots (presented in Figure 3 and Figure 4), as produced by the MANOVA procedure, which controls for the influence of the second dependent variable, showed the following:

- Teachers from Chile (ABA1), Australia (AUS1), the Flemish Community (Belgium-BFL1), Spain (ESP1), Netherlands (NLD1), Sweden (SWE1), and Viet Nam (VNM1) scored above the grand mean in job satisfaction.
- Teachers from Chile (ABA1), the United Arab Emirates (ARE1), Australia (AUS1), the Flemish Community (Belgium-BFL1), Denmark (DNK1), UK (ENG1), Netherlands (NLD1), Turkey (TUR1), and Viet Nam (VNM1) scored above the grand mean in self-efficacy.

The results from the job satisfaction profile plot cannot be compared with prior studies, as we were unable to identify any relevant studies that jointly compared teachers’ job satisfaction and self-efficacy across cultures for ISCED 1. Even the OECD TALIS 2013 results’ report [39] did not jointly investigate for mean differences among participants. However, in line with the evidence of Zieger, Sims, and Jerrim [19], UK teachers’ job satisfaction was significantly lower compared to other cultures. Further, our results corroborate with those of Klassen, Usher, and Bong [8], who found that Korean teachers had low job satisfaction. Regarding teachers’ self-efficacy these findings are aligned with the Vieluf, Kunter, and van de Vijver [9] and the Klassen et al. [10] studies. Specifically, Korea’s scores adjusting for the effects by job satisfaction were much improved as they were closer to the grand mean. Northern European and Anglophone countries, such as Denmark and the UK, were stable in their scores. Further, Spain had displayed, in compliance with the previous study [9], a low mean score. 

### 4.3. Implications and Limitations

To sum up, it seems that the multidimensional construct “school climate” has a significant impact on teachers’ self-efficacy and job satisfaction. This finding is suggestive of the necessary improvements that need to be made in order to promote job satisfaction through school climate. For instance, it could be said that the fostering of teacher-students’ relations and improving teachers’ perceptions of disciplinary climate in the classroom (e.g., students’ misbehavior) may contribute to raising teachers’ job satisfaction. Additionally, it is important to enhance teachers’ self-efficacy because the correlation (see Section 3.2 Results) between job satisfaction and teachers’ self-efficacy is statistically significantly positive, i.e., higher teachers’ sense of self-efficacy may result in higher levels of job satisfaction. In addition, as job satisfaction is connected with students’ performance, teachers’ wellbeing and retention (e.g., [7]) then by raising teachers’ self-efficacy we would be able to promote teachers’ wellbeing and resilience. Further, the cross-cultural comparisons, provided in this article, may enable psychologists to design the most appropriate intervention programs to enhance teachers’ sense of self-efficacy in conjunction with their job satisfaction. 

Lastly, as with every empirical investigation, this study has also some limitations. First of all, it would have been more indicative, if the complete databases were available in order to investigate for any additional effects among the variables under study and teachers’ job stress. Moreover, the current views were constrained by the restricted range of prior research literature on the topic. Further, regarding the research instruments, we believe that Model I, as presented in the Results section, indicated a negative indirect effect due to the negative intrinsic meaning of the teachers’ perceived disciplinary climate, but as a whole, it was conceptually sound. Thus, in a future replication of these results, a positively meaningful scale is recommended. Lastly, the TALIS data are cross-sectional and do not allow for a causal interpretation of the findings, because there was no temporal precedence established. In this study, it has not been feasible to investigate for latent mean differences (see Section 3.2). We recognize that state-of-the-art advances in SEM, such as the Alignment method [40], enable researchers to bypass the issue of scalar non-invariance, however, it is beyond our present capacity to implement that approach. Lastly, model fit has, also, not been taken into consideration at the separate country level, because the research has focused on the individual teachers’ level.

## Figures and Tables

**Table 1:** Distribution of the sample across countries.

Country	Frequency	Percent
Australia	3030	5.9
Flemish Community (Belgium)	2662	5.1
Chile	2514	4.9
Denmark	2592	5.0
England (United Kingdom)	2009	3.9
France	1429	2.8
Japan	3308	6.4
Korea	3207	6.2
Netherlands	1504	2.9
Spain	7246	14.0
Sweden	2404	4.6
Chinese Taipei	3494	6.7
Turkey	3204	6.2
United Arab Emirates	9188	17.7
Viet Nam	3991	7.7
Total	51,782	100.0

**Table 2:** Teachers’ self-efficacy scales.

**“In your teaching, to what extent can you do the following?”**
Self-efficacy in classroom management (CLMAN)
1. Control disruptive behavior in the classroom.
2. Make my expectations about student behavior clear.
3. Get students to follow classroom rules.
4. Calm a student who is disruptive or noisy.
Self-efficacy in instruction (INSTR)
1. Craft good questions for students.
2. Use a variety of assessment strategies.
3. Provide an alternative explanation, for example when students are confused.
4. Vary instructional strategies in my classroom.
Self-efficacy in student engagement (STUDENG)
1. Get students to believe they can do well in school work.
2. Help students value learning.
3. Motivate students who show low interest in school work.
4. Help students think critically.

**Table 3:** School climate scales.

**“How strongly do you agree or disagree with the following statements about this class?”**
Teachers’ perceived disciplinary climate (DISCP)
1. When the lesson begins, I have to wait quite a long time for students to quieten down.
2. Students in this class take care to create a pleasant learning atmosphere (-)
3. I lose quite a lot of time because of students interrupting the lesson.
4. There is much disruptive noise in this classroom.
Teacher-student relations (REL)
1. Teachers and students usually get on well with each other.
2. Most teachers believe that the students’ well-being is important.
3. Most teachers are interested in what students have to say.
4. If a student needs extra assistance, the school provides it.

Note: (-) Items were reverse scored.

**Table 4:** Job satisfaction scales.

**“We would like to know how you generally feel about your job. How strongly do you agree or disagree with the following statements?”**
Job satisfaction with work environment (ENVR)
1. I would like to change to another school if that were possible (-)
2. I enjoy working at this school.
3. I would recommend this school as a good place to work.
4. All in all, I am satisfied with my job.
Job satisfaction with profession (PROF)
1. The advantages of being a teacher clearly outweigh the disadvantages.
2. If I could decide again, I would still choose to work as a teacher.
3. I regret that I decided to become a teacher (-)
4. I wonder whether it would have been better to choose another profession (-)

Note: (-) Items were reverse scored.

**Table 5:** Path coefficients, standard errors, and bootstrapping results [Model I].

Path	B Coeff.	β Coeff.	S.E.	B Bias S.E.	B Bias (Bias 90% CI)
TSES←CLIM	1.13 ***	0.56	0.02	0.00	[1.08; 1.19] **
SATIS←TSES	−0.18 ***	−0.28	0.01	0.00	[−0.21; −0.16] **
SATIS←CLIM	1.25 ***	0.92	0.04	0.00	[1.17; 1.32] **
CLMAN←TSES	0.84 ***	0.80	0.00	0.00	[0.84; 0.85] **
INSTR←TSES	0.87 ***	0.83	0.00	0.00	[0.87; 0.88] **
STUDENG←TSES	1.00	0.90	−	0.00	
DISP←CLIM	−0.79 ***	−0.35	0.01	0.00	[−0.82; −0.77] **
REL←CLIM	1.00	0.55	−	0.00	
PROF←SATIS	1.00	0.55	−	0.00	
ENVR←SATIS	1.49 ***	0.91	0.02	0.00	[1.45; 1.53] **

Note: *** significant at *p* < 0.001; ** significant at *p* < 0.01.

**Table 6:** Path coefficients, standard errors, and bootstrapping results [Model II].

Path	B Coeff.	β Coeff.	S.E.	B Bias S.E.	B Bias (Bias 90% CI)
TSES←REL	0.26 ***	0.23	0.00	0.00	[0.25; 0.27] **
TSES←DISP	−0.21 ***	−0.23	0.00	0.00	[−0.21; −0.20] **
SATIS←TSES	0.06 ***	0.09	0.00	0.00	[0.05; 0.06] **
SATIS←REL	0.30 ***	0.40	0.00	0.00	[0.29; 0.30] **
SATIS← DISP	−0.06 ***	−0.11	0.00	0.00	[−0.07; −0.06] **
CLMAN←TSES	0.85 ***	0.80	0.00	0.00	[0.84; 0.85] **
INSTR←TSES	0.87 ***	0.83	0.00	0.00	[0.87; 0.88] **
STUDENG←TSES	1.00	0.90		0.00	
PROF←SATIS	1.00	0.55		0.00	
ENVR←SATIS	1.53 ***	0.92	0.02	0.00	[1.49; 1.57] **

Note: *** significant at *p* < 0.001; ** significant at *p* < 0.01.

## Appendix A

**Table A1:** Descriptive statistics for the composite variables under study.

	N	Min.	Max.	M	SD
Self-efficacy: classroom management	51,782	4.00	16.20	13.601	2.210
Self-efficacy: instruction	51,782	4.00	16.00	13.289	2.194
Self-efficacy: student engagement	51,782	4.00	16.00	13.240	2.323
Teacher-student relations	51,782	4.00	16.00	13.510	1.853
Teachers’ perceived disciplinary climate	51,782	4.00	16.00	8.3928	2.324
Job satisfaction with work environment	51,782	4.00	16.00	12.557	2.291
Job satisfaction with profession	51,782	4.00	16.00	12.414	2.507
Job satisfaction (composite)	51,782	8.00	32.00	24.971	4.168
Self-efficacy (composite)	51,782	12.00	48.20	40.130	6.065
Valid N	51,782				

Note: N: Sample size; M: Mean; SD: Standard Deviation; Min.: Minimum value; Max.: Maximum value.

**Table A2:** Goodness-of-fit indices for measurement Models I and II.

	CFI	TLI	RMSEA (90% CI)	SRMR	AIC	BIC	BCC
Model I	0.97	0.95	0.07 (0.073–0.077)	0.03	3253.10	3403.63	3253.10
Model II	0.99	0.99	0.01 (0.014–0.021)	0.02	86.32	183.73	183.73

Note: The interfactor correlations (φs) are all below the 0.85 rule (see [34]).

**Table A3:** Omnibus multivariate criteria.

	Value	F	Hypothesis df	Error df	Sig.	Partial *η^2^*
Pillai’s trace	0.360	812.07	28.00	103534.0	0.000	0.180
Wilks’ lambda	0.665	837.83	28.00	103532.0	0.000	0.185
Hotelling’s trace	0.467	863.74	28.00	103530.0	0.000	0.189
Roy’s largest root	0.365	1349.43	14.00	51767.0	0.000	0.267

**Table A4:** Descriptive statistics for job satisfaction and self-efficacy (mean comparison).

	N	M		SD		Min.		Max.	
Australia	3030	25.538	40.902	3.976	5.089	9.00	21.00	32.00	48.11
Flemish Community (Belgium)	2662	25.861	40.958	3.857	4.624	8.00	23.00	32.00	48.00
Chile	2514	26.502	41.576	3.482	4.724	11.00	12.00	32.00	48.00
Denmark	2592	24.757	40.806	4.123	4.447	8.00	12.00	32.00	48.00
England (United Kingdom)	2009	24.790	42.271	4.268	4.817	8.00	24.00	32.00	48.16
France	1429	24.684	36.156	3.890	5.366	8.00	19.00	32.00	48.00
Japan	3308	23.555	30.589	4.034	5.567	8.00	12.00	32.00	48.00
Korea	3207	23.806	39.230	4.641	6.394	8.00	12.00	32.00	48.00
Netherlands	1504	25.206	41.355	3.665	4.188	11.00	14.00	32.00	48.00
Spain	7246	27.328	39.156	3.536	5.278	11.00	15.00	32.00	48.00
Sweden	2404	24.989	38.261	3.957	5.149	10.00	18.00	32.00	48.00
Chinese Taipei	3494	23.226	38.261	3.459	5.499	8.00	12.00	32.00	48.00
Turkey	3204	23.673	40.911	4.398	5.701	8.00	12.00	32.00	48.20
United Arab Emirates	9188	24.167	43.425	4.437	5.222	8.00	12.00	32.00	48.00
Viet Nam	3991	25.469	42.478	3.166	4.739	14.00	21.00	32.00	48.00
Total	51,782	24.971	40.902	4.168	6.065	8.00	12.00	32.00	48.20

Note: N: Sample size; M: Mean; SD: Standard Deviation; Min.: Minimum value; Max.: Maximum value.
